# Towards an understanding of maltreatment in football

**DOI:** 10.3389/fspor.2024.1350317

**Published:** 2024-05-31

**Authors:** James A. Newman, James L. Rumbold

**Affiliations:** Sport and Physical Activity Research Centre, Health Research Institute, Sheffield Hallam University, Sheffield, United Kingdom

**Keywords:** safeguarding, abuse, power, mental health, disengagement, harm

## Abstract

**Introduction:**

This study explored the understanding of maltreatment from the perspective of various personnel working in roles related to safeguarding and welfare in English professional and semi-professional football.

**Method:**

Through a social constructivist position, the present study was able to explore multiple understandings and perceptions of maltreatment in football. Individual semi-structured interviews (*M*Duration = 68.00 minutes, *SD* = 9.05 minutes) were conducted with 19 participants working across league structures ranging from the English Premier League (EPL) to the English Northern Premier League Division One, as well as individuals working with some of the principal organizations in English professional football.

**Results:**

Through reflexive thematic analysis, three general dimensions were highlighted: “current understanding of maltreatment in football,” “constituents of maltreatment,” and “signs and symptoms of maltreatment.” Findings from those working in a safeguarding capacity mirror the research literature around understanding the components of maltreatment but also demonstrate how wrongdoing is nuanced by the football context, in that certain forms of maltreatment are driven by the unique nature of this environment.

**Discussion:**

From an applied perspective, the findings also outline how to distinguish both the more overt and covert signs and symptoms of maltreatment, whilst also highlighting the impact of maltreatment on individuals' mental health and their sense of self. Overall, the findings provide a platform for practitioners and researchers to consider in the design of safeguarding and welfare provision by highlighting the need to raise knowledge and awareness of maltreatment whilst intervening to challenge the prevailing workplace culture within professional football.

## 1 Introduction

In 2023, Hartill et al. highlighted the scale of the issue of interpersonal violence in sport through surveying 10,302 respondents aged 18–30 who had participated in organized sport prior to age 18. Specifically, 65% of those respondents reported experiencing some form of psychological violence in sport (1). Although professional football in the UK lacks similar data, the sport has been engulfed in similar reports of maltreatment, abuse, and bullying impacting players, coaches, and referees (2–5). This is especially concerning considering findings which suggest that maltreatment in the form of bullying leads to dropout from sport, as well as emotional harm and burnout in adult football participants (6–8). Despite significant attempts to understand maltreatment in contexts such as football, the plethora of terms (e.g., abuse, bullying, violence, and exploitation) and the interchangeable nature of their use has led to inconsistency in understanding, replicability, and transferability of research in this area over time (9, 10). Therefore, it remains of great importance to explore views of maltreatment in specific sport contexts, such as football given this environment has been noted for the justification of abusive and intimidatory actions (11) which drive and legitimize these forms of wrongdoing. Moreover, at present, safeguarding policies related to maltreatment are lacking in professional football and instead, only limited information is available that addresses certain aspects of this behavior such as online abuse (12). The result of this is that individuals' understanding, and experiences of maltreatment may vary even if they are working in a safeguarding capacity, which may impact the way wrongdoing is addressed. Thus, exploring the perspective of multiple individuals working in this capacity is critical.

### 1.1 Defining maltreatment

Various authors have considered the overarching term of maltreatment to encapsulate the numerous forms of harm which may occur in sport (10, 13, 14). Specifically, these authors operationalize maltreatment as:

all forms of physical and/or emotional ill-treatment, sexual abuse, neglect or negligent treatment or commercial or other exploitation, resulting in actual or potential harm to the child's health, survival, development or dignity in the context of a relationship of responsibility, trust or power (15).

Notably, though, these definitions emphasize maltreatment with children rather than adults, highlighting a potential lack of understanding specific to the adult population. Nonetheless, this research does provide important information about how maltreatment can be understood in terms of “acts of omission (e.g., harmful inaction), commission (e.g., harmful action), and exploitation (e.g., deceptive, cynical and harmful use of one person for another”s benefit)” (10). Moreover, this work highlights how maltreatment includes various forms of interpersonal violence and harm which can be seen as direct (e.g., from one individual to another), indirect (e.g., administered through others), intentional (e.g., with malicious intent) or unintentional (e.g., without malicious intent) (16, 17). As a result, maltreatment is made up of harm in the form of different types of abuse (physical, sexual, neglect, and emotional/psychological), as well as discrimination, institutional, and virtual maltreatment (17).

The findings presented thus far, align with the established conceptual framework for understanding maltreatment in sport (18). This model proposes maltreatment as an umbrella term that can be broadly categorized into relational forms (split into the four categories of physical abuse, sexual abuse, emotional abuse, and neglect) and non-relational forms (split into six categories of harassment, bullying, corruption/exploitation, sexual exploitation/prostitution, institutional maltreatment, child labor and abuse/assault occurring within a non-critical relationship with the individual). Stirling (18) suggested that relational maltreatment occurs in the context of a critical relationship in which one member (e.g., a coach) has significant influence over another (e.g., a player). Examples of other relationships in which these occur include parents and other athletes who may be in a mentoring role (e.g., team captains in football). By contrast, non-relational maltreatment occurs when an individual does not have direct influence over another (e.g., peer-to-peer bullying). Other relationships where these forms of maltreatment may occur include with an official, sport administrator or the sport organization. These forms of maltreatment may also be reinforced through external outlets such as the media (19).

To date, Stirling's (18) model remains the predominant conceptual framework of maltreatment in sport, although it is acknowledged that contemporary research has highlighted a plethora of other forms of harm which may be categorized as maltreatment (20, 21). This can involve direct physical harm such as punching, beating, and kicking, as well as indirect forms such as holding a position for longer than necessary*.* Equally psychological harm can be experienced in the form of belittling, denigrating, scapegoating, threatening, scaring, discriminating, and body shaming*.* Given the inconsistency in the terminology used to define harm and the variability in the sample examined in terms of the level of sport and age of participants (21), it is potentially unsurprising that findings have been mixed in terms of the reporting of maltreatment. As an example, research with adult professional footballers on their understanding of bullying demonstrates this (8). Players in this study reported that bullying occurs both within critical and non-critical relationships in contrast to Stirling's (18) frameworks of maltreatment. Part of this issue may be explained by the complexity of understanding maltreatment (22). This is further complicated by what has been described as the “grey area” of relationships, such as between a coach and athlete (14). “Grey area” behaviors may be deemed as either acceptable or unacceptable, depending on the circumstances, the intent and frequency of the potential maltreatment (14). The result of this are issues with the reporting of maltreatment which might be reflective of the degree to which key personnel (e.g., football players, coaches, sport scientists and welfare officers) are educated (or not) about this behavior (22). These findings are compounded by recent research which highlighted the volatile culture around reporting wrongdoing in professional football, that leaves players in fear of speaking out (23).

### 1.2 The sport/football culture

Alongside the challenges of understanding maltreatment, it is also important to consider whether the prevailing culture of sport and football is an issue. Inherently, individuals may be bound to what Hughes and Coakley (24) describe as the “sport ethic”, that prioritizes performance over wellbeing. This exacerbates the potential for maltreatment as beliefs that performance is a result of mental toughness, resilience and perseverance (25) become problematic. These beliefs coupled with a culture of winning at all costs, normalizing harm, a lack of equity, diversity, and inclusion, a culture of fear and silence, and a lack of trust in organizations to handle cases of harm (21) can further increase the potential for maltreatment. Furthermore, the centrality of the coach in determining a sport organization's culture (particularly if they are seen as successful) may also present difficulty (25), especially as these individuals can normalize emotionally abusive practices (26). Coaches' negative approaches can be ingrained, stemming from their careers as athletes and/or the experiences of other coaches, which, when coupled with situational factors such as job insecurity (27), can provide an additional context in which maltreatment can grow (28). The result can be a grey area within coaches' relationships with athletes, where the latter individuals often navigate maltreatment situations by accepting them through normalization (14). Unfortunately, other researchers have also shown that these practices of maltreatment extend beyond coaches to other members of an athlete's entourage, such as parents, partners, and general team managers (20, 28).

In football particularly, the abusive and intimidatory practices of coaches and managers (29) are potentially legitimized to an even greater extent with adult participants, threatening the safeguarding of this population in this context. Within the professional football environment, violent and abusive language, scornful humor and personal castigation are commonplace (30), reinforcing a “hidden curriculum” around the need to accept severe banter (31). Individuals are expected to give and receive insults to the point where others fail to cope and ultimately “snap” (30), despite more recent research revealing that this verbal abuse is seen as bullying (8). In common with research focusing on emotional abuse in sport (26), players were of the view that coaches may not be aware that abuse or bullying is taking place (8). Findings of this nature are indicative of the potential issues of a lack of understanding and awareness of maltreatment from key personnel (e.g., coaches) in sports such as professional football. Thus, in recent times there has been some gradual movement towards exploring the perspective of personnel with an interest in advancing safe sport (32–34). While this movement has made encouraging advances in understanding the perspective of those in leadership and administrative roles in elite sport, as well as other key personnel such as sport psychology consultants, these studies have not directly explored the perspectives of those directly tasked with safeguarding and welfare (e.g., such as heads of safeguarding/welfare). Engaging such personnel in football is critical, as at present, policy frameworks regarding the safeguarding and prevention of maltreatment are limited to focusing on children and vulnerable adults and often do not explicitly cover maltreatment itself (35, 36).

### 1.3 The present study

Through specifically focusing on staff in football with a responsibility for safeguarding and welfare, the present study answered calls for research to incorporate interviews with policymakers and others involved in the policy-making process (37). Moreover, the study took a unique approach to understanding maltreatment, by exploring football personnel's (e.g., chief executive officers, player care leads, safeguarding leads, and education/welfare leads) perceptions of this concept concerning non-vulnerable adults rather than children or vulnerable adults. Specifically, the central research question guiding this qualitative inquiry was what are football personnel's (e.g., chief executive officers, player care leads, safeguarding leads, and education/welfare leads) understanding of maltreatment? Drawing on a co-design approach which drew on the participants' experiences and knowledge, the present study enabled key personnel in football to co-design the findings in partnership with the researchers (38), to improve the understanding of maltreatment in football.

## 2 Materials and methods

### 2.1 Research paradigm

Given this study involved exploring football personnel's perspectives on maltreatment and safeguarding, a social constructivist position was adopted. The use of a constructivist lens was ideal in focusing on the multiple understandings and perceptions (39) of football's various personnel. Grounded in a relativist ontological viewpoint, the study was guided by the view that there is no singular truth and instead, multiple realities exist as meaning, interpretations, and experiences in relation to maltreatment in football differ between participants (40). For example, through adopting this lens, the present study was mindful that views on maltreatment in football may be shaped by the context of the club the participant was working at and the participant's previous experiences of working football, as well as other occupations. In terms of an epistemological stance, the constructivist approach regards the researchers and participants as being active in the co-construction of knowledge as part of its subjectivist/transactional view (40). Moreover, this research approach has been applied within studies of safe sport, as part of a co-dependent process between researchers and participants (32). This was seen as well-matched to this study's approach of developing findings between the participants and researchers through co-design. Therefore, to maintain consistency with other co-design approaches the present study drew on the experiences and perspectives of the participants through interviews (38). Key points concerning maltreatment were identified by the football personnel and were prioritized by the researchers in the development of the findings, in combination with evidence from the literature in this area (38).

### 2.2 Participants

Semi-structured interviews were conducted with 19 participants (*MAge *= 44.21, *SD *= 10.03, range = 28–70 years). This approach offers the benefit of structuring questions based on previous research on maltreatment [e.g. (34),], whilst offering flexibility to the participants to guide the interview around areas of interest and importance concerning this concept. The sample size was determined following recommendations for qualitative research, whereby sufficient participants were recruited to tell a rich, complex, and multifaceted exploration of maltreatment (41). The participants were recruited from clubs ranging from the English Premier League (EPL) to the English Northern Premier League Division One, as well as the principal organizations in English professional football (further details on the recruitment process are provided in 3.3 Procedure). At the time of interviewing, the participants held a range of appointments in football including Chief Executive Officer, Vice Chairman, General Counsel, Club Development Officer, Head of Safeguarding, Designated Safeguarding Officer, Safeguarding Case Officer, Academy Safeguarding Manager, Head of Education and Welfare, Player Care and Welfare Officer, Head of Education and Player Care and Coach. By focusing on members of staff who are specifically responsible for safeguarding and welfare, the study extended beyond the perspectives of administrators [e.g. (32),] in order to understand maltreatment further within the context of elite sport. To this end, by drawing on the perspectives of safeguarding and welfare personnel in football, combined with the extant research evidence it was hoped that a more detailed conceptualization of maltreatment could be provided (33).

### 2.3 Procedure

Following ethical approval by the University Research Ethics Committee [ER41451626], the participants were initially contacted via a combination of emails and LinkedIn messages. Initially, this process involved convenience sampling focusing on participants who were known/accessible to the researchers and who met the criteria to participate in the study (32). To be eligible participants had to be non-vulnerable adults, working for a professional/semi-professional football club in either a designated safeguarding role or a position in which safeguarding was one of their key responsibilities. As the study progressed a mixture of purposeful and snowball sampling was used to identify participants who were consistent with the trajectory of the study (32). Once the potential participants had indicated a willingness to take part, they were provided with an information sheet which provided additional details regarding the study as well as a consent form which was signed before the interview commenced. Furthermore, as the participants held positions of responsibility they were reassured that confidentiality would protect them from being perceived negatively and would also safeguard them from jeopardizing their position within their organization (32). To assist in this process the participants' names and their respective organizations were replaced by pseudonyms.

Once full consent had been provided semi-structured interviews were conducted by the first author with each participant to explore their understanding of maltreatment. Interviews lasted between 54 and 83 min (*MDuration* = 68.00, *SD* = 9.05). The interviews began with rapport-building questions, such as “How are you?” and “Can you describe a typical day or week in football?” Then the interviews progressed towards questions around maltreatment “Could you tell me in your own words what maltreatment means in football?” and “What sort of effects would maltreatment in football have?” This semi-structured interview guide was developed from the existing literature on maltreatment in sport (34, 42). It should be acknowledged that due to the socially constructivist position adopted, the interview guide was based on a mixture of pre-determined questions and those which were adjusted to the individual and their football context (39). Specifically, this guide was amended where required to address areas of interest raised by the participants (39), and space was allowed to take into account unanticipated directions to the questions and answers (33). Finally, the interviews were conducted via Microsoft Teams. All the interviews were audio recorded and then transcribed verbatim using a combination of Microsoft Teams' transcription software and manual transcription, before being analyzed. All data for the present study was managed in line with United Kingdom General Data Protection Regulation (GDPR) 2018.

### 2.4 Data analysis

Data were analyzed for themes in participants' accounts using reflexive thematic analysis [RTA (43);]. RTA is described as a unique approach to thematic analysis in that it acknowledges and values the researcher's role in knowledge production whilst offering the theoretical flexibility to fit this study's social constructivist stance (43). Given RTA's potential to conceptualize patterns of shared meaning around a central organizing concept (44), it was seen as ideal for developing an understanding of maltreatment in professional football. The analysis did not progress in a linear fashion, and instead involved a recursive journey back and forth between stages (45, 46).

The thematic analysis steps employed in the present study revolved around those set out by Braun and Clarke (47). Firstly, after the transcriptions were complete, the lead author familiarized themselves with the data by reading and re-reading it. Secondly, the authors went through a process of systematically analyzing and interpreting meaningful segments of text to understand views of maltreatment, adding labels to generate initial codes (46). Once these codes were developed, they were reviewed, combined, and interpreted in terms of shared meanings to form themes (46). A process of thematic mapping was employed at this point (45) to make sense of and connect patterns of shared meaning, review themes, define and name themes and produce the report. For example, themes were generated around the different types of abuse which make up the constituents of maltreatment. Once this process was complete across the themes the second author challenged and suggested alternative perspectives on the themes through the process of being a “critical friend” (48). Drawing on “critical friends” was important in the present study as it is a hallmark of rigor in qualitative research where a relativist rather than criteriologist position is adopted (49), therefore researchers do not necessarily need to agree in their views of certain themes (48). The themes were then further analyzed, defined and, written up (46).

It should be noted that due to the inductive approach taken, themes were generated in the participants' rather than the researchers' language. Therefore, given the investigators' experience in publishing scientific research in areas relating to maltreatment, care was taken to ensure that data was not forced into preconceived categories (50). Once this process was complete the themes were then reviewed via deductive reasoning (51). This aided with the categorization of findings into higher-order themes and general dimensions based on research literature and theory (51).

### 2.5 Research quality

For this project, a relativist position was adopted to ensure sound qualitative practice and to maintain data trustworthiness (23, 48). As a marker of quality, the underlying philosophical assumptions were set out for the study as a means of illustrating how theoretical assumptions influenced the study design, analysis, and the authors' relationship with the participants (52, 53). Moreover, a reflexive approach was taken (8), whereby both authors identified their positions as researchers who have published research in areas associated with maltreatment (e.g., bullying) in professional football. The first author also has experience of working as a psychology practitioner in professional football. Finally, both authors identified their positions as fans of professional football. As such both researchers carefully monitored their presuppositions regarding the football context (8).

Further consideration from qualitative research in sport (48) was made to develop rigor within the present study. Firstly, credibility was ensured within the data by providing rich and detailed descriptions from the participants. Secondly, ethical considerations were met by providing a “sensitivity to the context” (54) in terms of protecting the anonymity and confidentiality of the participants due to the nature of the content discussed and the potential vulnerability of their roles, given the potential organizational issues in sport more broadly. Finally, the study also aimed to provide naturalistic generalizability (55), such that it resonated with the reader's personal engagement or vicarious experiences of maltreatment in professional football.

## 3 Results and discussion

Analyses of the participants' data revealed three general dimensions. These dimensions *the current understanding of maltreatment in football, the constituents of maltreatment, and the signs and symptoms of maltreatment* presented some similarities with existing conceptual models of maltreatment in sport [e.g. (18),], whilst extending understanding of this concept within the professional football context specifically.

### 3.1 The current understanding of maltreatment in football

Across their accounts, the participants evidenced how maltreatment is understood in professional football (see Table 1). This was underpinned by the higher-order themes of producing players and succeeding, transcending football, lack of knowledge and awareness, football cultures affecting understanding, and the power dynamics at the heart of maltreatment.

**Table 1:** The current understanding of maltreatment in football.

Example raw data code		Lower-order theme		General dimension
The importance of winning	→	Producing players and succeeding	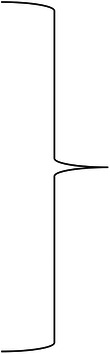	Understanding
Part of society/life	→	Transcending football		
Unintentional behavior	→	Lack of knowledge and awareness		
Misguided perceptions	→	Football cultures affecting understanding		
A fear of speaking out	→	Power dynamics at the heart of maltreatment		

#### 3.1.1 Producing players and succeeding

For some participants, maltreatment was described as a necessary means to an end that was driven by the wider context of the club and systemic culture of professional football:

It sustains business models, it achieves funding. So you're a Cat(egory) One Academy and you're receiving £2 million a year because you're hitting standards and producing players again. The whole concept of football is (…) to win (…) and to win, it could mean win at all costs, and the win supersedes [everything] (…) If you're a coach (…) your job isn't just to look after that player, it is to is to score more goals than the other team. You know and (…) it can be that really that simple. (Giles).

Giles highlighted how the pressures both systemically in terms of receiving funding, and the importance of winning, drive a feeling in individuals that maltreatment may be necessary to hit these standards. This drive disregards players' welfare at both a systemic and more localized club level, where norms and values around a “will to win” (11) supersede safeguarding concerns. Furthemore, at the relational level, Giles described, how “discipline and nurture can be competing needs” for coaches, which results in the needs of an individual being “overridden over and again.” The result is a “trickle-down” effect of maltreatment where coaches culturally reproduce and accept acts of wrongdoing that they experienced in professional football as part of football's hidden curriculum (31).

The sense that maltreatment is “necessary” spread into views of how players are treated when they were not seen to make the grade for a particular club. Cheryl illustrated this, “there's a situation I’d say around releasing people. And I suppose they [coaches] have to some extent be OK with it, because they have to do it and it's a really difficult thing to do.” Interestingly, although Cheryl appeared on one level to highlight the problematic nature of this perception in professional football by placing the spotlight on this “rationalization,” she also started to show how this view interacted with her own psyche by rationalizing that some individuals “have to” behave in this way. Again, the need to succeed appeared to move beyond the need to protect individuals.

#### 3.1.2 Transcending football

Although the general tendency was for the participants to situate their understanding of maltreatment within the football context, others highlighted how this concept transcends football. Indeed, for Michael, maltreatment functions at a broader systemic level,

I don't think it's necessarily just in football though. I think it could be any industry that you're in (…) It got a lot worse for me when I was in my previous role as safeguarding, designated safeguarding lead at a primary school. And having to go to mapping meetings.

Michael's view demonstrated broader concerns about maltreatment at a societal level which feeds into football, reflecting findings in other industries (e.g., education) where there is a tendency to focus on academic achievement rather than how an individual is functioning in the school environment (56). Thus parallels can be drawn between the performance-oriented nature of professional football (57) and school achievement where maltreatment often breeds from these pressures, and safeguarding concerns arise. As such, the findings add important information about the potential risks of achievement-based cultures in football and society.

In furthering their account, Michael discussed how maltreatment is also a product of normal human functioning, though he did suggest that individuals can make attempts to address this behavior “I know this is about football, but I don't think necessarily just football that this relates to. I think we're all sort of, we're all humans.” Other participants described a different view of how maltreatment transcends football to the extent that it might not be seen as applicable in this context. When asked to conceptualize maltreatment in football, Anna said,

Maltreatment is not a word I use. I'm gonna be honest, I don't use it. Maltreatment to me implies a (…) and so, gosh, I would hope in football that that's not, that's not the case. I'd hope in any kind of organization.

In this case, there was almost a sense of disbelief that maltreatment might exist in football. The pause in Anna's quote around the implication of maltreatment alluded to a sense of the severity of this concept yet was also revealing a (dis)belief that this could occur in football. As such this revealed the gravity of this term but potentially also a sense that professional football as a culture would be shocked if it occurred in this context.

#### 3.1.3 Lack of knowledge and awareness

A striking finding from across the participants revolved around the lack of knowledge and awareness of maltreatment. For Kyle, this lack of awareness is fueled by naïve beliefs of individuals, leading to maltreatment,

I do also think that you can make honest mistakes and we're all educated differently, we're all brought up differently and what you're taught is appropriate language (…) we had to get a player to take a Tweet down a couple of weeks ago because he'd waded in with his opinion on the abortion decisions about the Supreme Court in the US. And his beliefs weren't in keeping with those of the majority of XXX supporters. And I just felt that as somebody representing the football club, it wasn't really appropriate for him to share his political opinion on something that could be deemed so offensive to a lot of people. So I'm sure he wouldn't have said his opinion was banter, but his opinion is different to everybody else's because of his own faith, his own culture, his own education. But that's not to say that it's OK to share it.

This reveals the challenges faced around developing an understanding of maltreatment in professional football. On the individual level, Kyle outlined how the person concerned made an honest error that was not in keeping with the club's standards. Yet within professional football, this can be challenging, due to the multi-cultural nature of this environment, which may shape a vast array of views around the appropriateness of behavior.

The potential lack of education around maltreatment does not occur due to the country of origin alone, however, as Robert stated,

I'm not sure that there are that many individuals, certainly on the coaching, backroom side, that would have that much experience or knowledge of maltreatment. I suppose if it was something obvious like bruising, particularly to the face or something, which probably wouldn't have been caused in football itself, that might set alarm bells ringing. But I'm not sure football practitioners are that adept at dealing with the more emotional sides of maltreatment.

Quotes such as this exposed a critical gap in knowledge around maltreatment and safeguarding in football, with the findings reaffirming that coaches appear to lack sufficient skills and knowledge to understand this concept (58) and provide a duty of care to their athletes. Though it should be noted, as Kyle outlined previously, that the participants also supported the findings that this lack of awareness and knowledge spreads beyond coaches to other individuals such as players, who may be the instigators of abuse (59).

One explanation for a lack of awareness about wrongdoing in football may be explained by beliefs that maltreatment is only understood in terms of the significant cases of sexual abuse (57). While not directly corroborating this view, Gemma did highlight the problem of when more “accidental” forms of maltreatment may be occurring,

A lot of these complaints and things are unintentional and it is just a learning need. Or somebody's held a view or a way of working for a very long time, because, like I was talking about earlier where people have been in the system or their role for quite a long time.

It appears that the grey area of behaviors (14) may be shifted, such that more serious forms of abuse are either missed or not processed in football, due to both systemic issues around acceptance, as well as an individual's position in their role. Therefore, the results reinforce the need for intervention programs to educate those across the sport, particularly for the forms of maltreatment which may subtly cross the line between appropriate and inappropriate behavior (49, 60).

#### 3.1.4 Football cultures affecting understanding

For many participants the primary way they understood maltreatment was as a cultural issue in football. Simon's account was particularly reflective of this,

Once you go into the first team it's just a murky world and it's so dependent on the individuals that are in charge [leadership roles] at that time. This [interview room] door is not quite closed, so that's something to explore in itself. My level of comfort about talking with an open door, well everybody calls the manager gaffer, what century are we in here? There are ways to address people and ways to certainly not address people, an expectation about who you ring and what time you ring and how you interact with other people, and these are just implicit, you know, it's just stuff that's in the air and people just know. And if you fall foul of that, flipping heck, it's a tough school.

This account demonstrated the complex nature of the football context, where interactions with certain individuals are dangerous, and the assumed nature of what can be said and how individuals must behave comes with potential ramifications for those who transgress. Indeed, the concern expressed by this participant at the time of their interview, that their views may be overheard, spoke of a systemic level of fear. Such fear was indicative of a sense that concerns held by players that they will jeopardize their careers through reporting maltreatment (57), extends to other personnel within clubs. Thus, wrongdoing is sustained.

The systemic cultural issues around maltreatment extend beyond the playing staff to include those tasked with the responsibility of addressing inappropriate behavior. As Sarah described,

So prior to that I'd worked in … the public sector so there is lots of structure that is put in and you have an understanding of what's expected of you. But I think it's a very different culture within football and I think people have historically held a lot of power and I think the difficulty is, who provides the checks and balances? I think it's certainly better than it was, but it is not where it needs to be, and I think there is potential for if somebody is on a [executive] board or if somebody is a manager or is first team coaching staff. There are certain positions within the club that have the potential to not have those checks and balances applied appropriately because people are fearful. If you're me, if you're [job role excluded for anonymity] and the problem is the director, how do you go about that?

Several points were made here about the deeply ingrained nature of potential maltreatment. Any understanding that is present is controlled by certain individuals and a culture of fear persists both within the game more widely, as well as at the club level for individuals who need to challenge inappropriate behavior. Although some positive steps were highlighted, the necessary agency for individuals to check and challenge still does not appear to be available and welfare staff are left marginalized (57).

Furthermore, the cultural issue around maltreatment in football is problematic to the extent that cases of inappropriate behavior can become severely compromised,

I do think culturally where there's an allegation against somebody, everyone, what I've seen sometimes and particularly in the elite environment is staff around them will start taking a view. It's a bit like, “he would never do that”, “this is ridiculous”, which is quite dangerous, and it does isolate [people]. It makes the safeguarding team's job really difficult, particularly managing relationships after an allegation. (Robert).

These were quite concerning allegations about how individuals exercise their role as a bystander by offering personal opinions which may mean that inappropriate behavior is not addressed. As such it reveals the persistence of a culture of organizational bystanding in football (23, 61) where concerns around behavior are suppressed or never reported. Moreover, it suggests that the first instinct may be for individuals to inappropriately protect each other, due to the “intense loyalty” demanded by their clubs (23), rather than to work with other parts of their club/organization to handle cases professionally.

In a similar vein, the participants discussed how the understanding of maltreatment in football is currently shaped at the individual level. For example, this behavior is influenced by misguided perceptions, fueled by a lack of awareness around what maltreatment is:

You know somebody might even jokingly say to me “oh shut up baldy, fatty” or something like that. I'd shrug it off because I'm bald and I'm fat (…) but the point is that I can shrug it off and I’m not really bothered about it. But there are some people that might not be able to do that so what's maltreatment to one [person] is totally different to another. (Myles)

This quote demonstrated the dangers in perceptions around maltreatment in football, in that terms which may be upsetting or abusive to some individuals are used in the belief that they are ok. It also implies that the understanding of this concept is driven by the individual perpetrator's perspective and that victims must “shrug off” these forms of abuse.

#### 3.1.5 Power dynamics at the heart of maltreatment

For some participants, one of the main ways to understand maltreatment in football was through the power dynamics which sit at its core. These were seen as particularly central in illuminating this concept,

There's a lot of power in football and that creates silence. And one of the biggest things you see in football from a tiny age all the way up is that nobody wants to do anything to jeopardize their on-field time. So they will experience behaviors or situations that they should never put up with, they should never tolerate, they should always speak out about, but that fear that it's going to impact their football is more powerful than whatever they're on the receiving end of. (Claire).

This portrayed a worrying account of what individuals are on the “receiving end” of in terms of their interactions, as well as broader concerns about the dangers of speaking out. It also illustrated a vulnerability players experience around their playing time, where a code of silence is maintained in football (62) which keeps maltreatment suppressed. Seemingly there is an unquestioning acceptance of subordination on behalf of the players to their managers and coaches (31).

This issue was compounded for Lucy who described footballers as “not employees in the same way I’m an employee.” For this participant, there was something around the affluence achieved by certain individuals which can breed potential maltreatment,

There is something around the power imbalances that exist that can sometimes cause the gaps for those behaviors that would fall under maltreatment to exist. So, there is something around that. There is something around salary and there's something around how we work.

Although this is not the only way in which a power imbalance can be achieved it was potentially illustrative of the power afforded to or gained by certain individuals, and the potential for this to drive maltreatment. Thus Lucy's point reaffirmed previous findings in relation to bullying [e.g. (8),] where financial status can be one of the hallmarks of the power which drives wrongdoing.

### 3.2 Constituents of maltreatment

The themes outlined by the participants in relation to their understanding of football provided the backdrop for identifying a range of factors which made up the constituents of maltreatment (see Table 2). This included commonly reported elements such as abuse, bullying, individual and institutional neglect, and discrimination, as well as components which are more nuanced to the professional football environment such as creating unpleasant environments, control and power over others, and commodification.

**Table 2:** Constituents of maltreatment.

Example raw data code		Lower-order theme		General dimension
Bullying	→	The common components of maltreatment	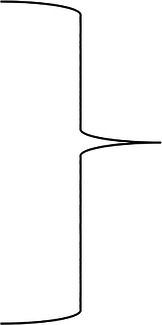	Constituents
Thwarting autonomy	→	Creating unpleasant environments		
Exploitation	→	Control and power over others		
The loans system	→	Commodification		

#### 3.2.1 The common components of maltreatment

Several of the participants viewed maltreatment as an umbrella term which covered various forms of abuse. Layla demonstrated this,

So, if you're looking at it from the outside in, and you're looking for signs of maltreatment you're going to be looking for a form of abuse. So, whether that's emotional, mental, bullying, coercive control, it brings us back to the money, you know.

Interestingly, Layla's account not only identified a range of commonly identified constituents in relation to maltreatment but also echoed the points made around how maltreatment is understood in football in terms of how money underpins power differentials. This reinforced a contextualized view of maltreatment in professional football which mirrored findings in relation to bullying (8). Furthermore, in listing bullying as a form of abuse Layla showed the potentially interchangeable use of these terms in contexts such as professional football. This finding was reflective of the participants often drawing on many of the constituents of maltreatment proposed by Stirling's (18) conceptual framework, yet it highlighted that the terms were used more interchangeably than in this model.

Although there was a tendency for several participants to collapse bullying into a discussion of abusive behaviors which underpin maltreatment, there were other occurrences where this concept was discussed for its standalone contribution as a form of maltreatment. Anna's account was indicative of this,

I guess for me, maltreatment would be kind of bullying (…) whether that's players, (…) on play[ers] it's like amongst players. Whether that's staff to players, you know coaches have all different coaching styles and you know I have no doubt that there are occasions where some overstep the mark, or it can seem quite targeted, or you know it's inevitable.

Within this extract, there were several notable points. Firstly, bullying is a strong component of maltreatment. Secondly, it is important to highlight that Anna viewed bullying as occurring across multiple relationships within football's hierarchy. This finding is noteworthy as it contrasts the conceptual model of maltreatment (18) where bullying is viewed as occurring within “non-critical” peer-to-peer relationships. Here Anna described a more fluid view of bullying which can occur across different levels of power relations. Finally, Anna's quote also highlighted the targeted nature of bullying.

Other participants though, highlighted the grey area nature of bullying in the form of banter and how this may manifest itself,

I think banter is an acceptable word to describe behavior that isn't acceptable on the whole. I think anything, if people are oversensitive or if people are offended by something the natural response is oh it's just banter, it's just a joke, you're too sensitive, you’re too this and you're too that. So it creates this safety net for those people who are intent on causing maltreatment and to kind of hide behind as well. (Claire).

Even though research demonstrates the potential for bullying through one-off acts (8, 63), these accounts were particularly illustrative of how behavior masquerades as “banter” and may hide bullying and wider maltreatment in football. Although these participants suggested that the use of banter at the individual level of relationships warrants consideration, there appear to be wider concerns about the social acceptability of this term and by proxy maltreatment within the football context. Thus, banter also needs considering as a form of bullying to expand upon conceptual models of maltreatment (18).

Common with the view of banter overlapping into bullying, some participants discussed how discriminatory language can also be excused through this term, when ultimately discrimination is a further indicator of maltreatment,

I think language is quite a tricky one because there are a lot of different people from different backgrounds, different faiths, different cultures and what that person from that background finds acceptable will be different to what you or I might find acceptable. And if I think that joking about a particular topic is my way of dealing with it, it may not be another person's. So I think that it is something where people can be cruel and then hide behind the term of banter. (Kyle)

This was a further representation of the potential systemic issue of the use of the term banter to mask the discrimination which forms maltreatment. These results can be understood from the historically masculine contexts of professional football where behaviors which are deemed to “cross the line” of acceptability in other sports such as cricket (64) are still accepted. Specifically, banter provides a legitimization to be “cruel” as Kyle described. It should be noted though that Kyle also drew reference to individual interpretations of appropriateness, which provided an important reminder that football's personnel have some responsibility over their behavior in this regard.

Aligned with the common identification of abuse, bullying, and discrimination as constituents of maltreatment, the participants highlighted neglect as another component of this concept. Alice outlined how this neglect functions on both a psychological and physical level, resulting in harm in both cases.

[Maltreatment is] not caring or respecting the athlete along with the person, I think, and so anything that that causes, (…) that person any (…) maltreatment will probably be significant things like neglecting their needs. Or you know, not giving them the best chance of fulfilling their job role and what they need to be doing etcetera. So yeah, probably say, you know, anything that causes them any sort of, (…) physical or psychological harm.

This account was indicative of occurrences where individuals are maltreated within the football context, whereby both their physical and psychological needs go unmet. Although this pointed to maltreatment occurring at the individual level, other accounts showed that these forms of neglect may be more driven at a wider club or systemic level, suggesting that the interaction of these factors leads to needs not being met. Unfortunately, this may reflect a failure such that safeguarding programs directed at the systemic level do not necessarily change individual behavior (14). As Anna described,

We're coming to the end of the season now. We're managing a lot of [player] release decisions. So like player welfare beyond when they're [contracted] with us and how we manage that as an organization and how we (…) support them to process that kind of thing. It's about the responsibility (…) that we have towards (…) our players that have been with us since 8–9 years old and then hit kind of 23 (…) and they're released (…) and I think if we're not doing that properly and we're not providing that support then for me that would come under maltreatment as well.

Context-specific challenges for football clubs were evidenced here around unfortunate decisions which need to be made with releasing players. Despite recommendations around proactive approaches to try to manage player welfare and transitions out of football (58, 65), the participants still highlighted the potential for maltreatment when players are released. Therefore, as Anna stressed, ownership needs to be taken by both clubs and the individuals within them to safeguard against maltreatment.

#### 3.2.2 Creating unpleasant environments

Considering the participants' identified forms of institutional neglect, it is potentially unsurprising that the football environment is one which at times is characterized by unpleasantness in terms of promoting false views around resilience and thwarting autonomy. Lucy captured this,

It's something around the systems and the processes and how they're set up so that we don't always give adults or adults at risk much autonomy in their decision-making. And that can lead to situations or circumstances where they feel disempowered. And for me that would probably come under a very large umbrella of maltreatment (…) I think that's an interesting way to think about maltreatment because you're talking about young people who could be earning thousands of pounds a week, but I think they are vulnerable, and I think they can be vulnerable in all sorts of different ways.

Lucy revealed an interesting dichotomy which connected to the previous theme around neglect. Although wealth gave players power, she described how money can leave footballers in a potentially precarious position which might make them susceptible to maltreatment (66). Moreover, despite this wealth individuals can be maltreated by their clubs through their autonomy being thwarted and their sense of self becoming devalued. This finding extends the parent-child literature which has found low autonomy support is a strong predictor of individual maltreatment (67). Within an adult group specifically, they revealed a contextualized view of maltreatment that is shaped by the football context.

Thwarting autonomy (68) was not the only factor that can occur at a club/systemic level which might lead to maltreatment. Several participants characterized a false view around resilience which creates unpleasant environments:

A lot of maltreatment will be treating younger people and young adults differently through trying to make them resilient to the rigors of professional football. And frequently I have heard this young man needs to toughen up. He could find himself in front of thousands of supporters baying for his blood, so therefore we wish to condition him almost. (Giles)

These is a notable finding both in extending the conceptualization of maltreatment in sporting contexts, as well as illustrating some of the common myths around resilience purveying the professional football context (69). As Dave summarized, this comes with a risk “they think they are developing resilience or pushing people, but it can, you know. I believe (…) it comes, there's a close line.”

#### 3.2.3 Control and power over others

As several participants noted across the general dimension of the constituents of maltreatment the professional football context is one in which vulnerability can be facilitated in various ways. One avenue for this is through the exploitation of individuals, as Sarah outlined,

Our players move up and down the country, there's not to say that anybody couldn't put any pressure on them to be moving substances around shall we say and being subject to those kind of pressures. Also in terms of modern slavery, when we talk about football and it's kind of been addressed on a few levels, but that's not to say that it still wouldn't happen. Obviously, we get approaches from people all around the world and approaches from agents all around the world and we're acutely aware that both young people and adults can be trafficked in a football context.

Sarah demonstrated that broader-level societal concerns such as modern slavery can infiltrate the professional football context through a football career being mis-sold as a viable strategy to lift individuals and their families out of poverty (70). The potential consequence is forms of maltreatment that may not be obvious to those outside of the sport. Therefore, professional football needs to be vigilant to these risks.

Maltreatment in the form of exploitation can spread beyond those working for a football club to the wider system of the sport, as Kyle experienced,

There are people out there that are motivated financially to do things that will hurt our players. We had a recent one where somebody was tweeting allegations against one of our players and you have to take them seriously, you have to contact the right bodies and you have to speak to the player as well and do your own internal investigations. But just one or two Tweets can really, particularly in professional football, can be really damaging and that individual is a young man and we've got to check on him as well.

This was particularly revealing of the challenges facing both football clubs and players in relation to maltreatment. In the first instance, this can leave individuals vulnerable to allegations that they have perpetrated maltreatment, yet they may be the ones who are victims. Significant challenges are posed here for both the individuals, the club, and football at large.

Nonetheless, there are elements where control and power are exercised by both clubs and individuals within them. Alfie discussed this, “You get that favoritism side of things and again I think a lot of it is false promises and letting people down, I class that as that's maltreatment.” Favoritism was a specific reflection of where the football environment actively maltreats individuals, echoing a feeling that players have to follow the coach's wishes due to fears around career progression (11). Furthermore, a reflection of how individual relationships in football can lead to maltreatment was offered by Simon,

A boy last year who's still with us, … he made the grave mistake of ringing the manager the night before a game to ask if he was in the squad, he's a first-year professional and he has to ask the manager if he's in the squad, he's not even been told. And ever since then the manager has just completely blanked him, wants nothing to do with him, you know, won't include him in training sessions, won't even look him in the eye, wants absolutely nothing to do with him, all because of a phone call at night. And this is an 18-year-old boy who is just desperately trying to make his way in the game.

This quote revealed a different side to how control and power are demonstrated by coaches and managers. Rather than more overt forms of bullying and abuse, ostracism can be used as an active, indirect strategy for maltreating individuals.

#### 3.2.4 Commodification

At the broader contextual level, the participants highlighted a systemic institutional view of maltreatment, specifically in relation to the commodification of players:

So we look at kind of the loans element, the way we're loaning young people out. You look at their relationships with their families, their relationships with their agents in particular [this] is really interesting, their peer group, the fact their friends can change overnight, constantly being I suppose judged on performance, you know, must be incredibly draining. We do a lot of work around mental health and resilience and wellbeing, and that is very much embedded in XXX, and it's embedded across all the clubs, but fundamentally we’re here to make money. (Lucy)

This quote was illustrative of a less obvious view of maltreatment in which players are treated as tradeable commodities who may become isolated from their families and peer groups quickly whilst being constantly subject to evaluative pressure. The quote also highlighted the perils of the loan system where treating individuals as financial assets can be extremely damaging to them. It would appear that gaps in the football system around preparing individuals for loans and not treating them individually (71) leaves their wellbeing uncared for and creates the conditions for maltreatment.

Anna elaborated on how the potential pressures of the football industry provide a breeding ground for maltreatment,

That in and of itself is awful … That's an awful concept, isn't it? This person could potentially earn us lots of money, … the thing is, again, … that's not a criticism of the of the organization. That's the reality of football. That's the reality of elite football is that they need to make money off their players to be able to be successful within the football industry.

Here the tensions of the football industry were exposed such that even an individual who is in a role to safeguard players presented the argument for how clubs rationalize the treatment of players. It also showed the contextual backdrop for how maltreatment may be produced.

Although many highlighted the importance of considering the commodification aspect in relation to players it is important to close this theme with a focus on all individuals within football. As Laurence cautioned,

So for example in our industry, the media side of the game. The number of media students that are leaving that will do anything to work in football is significant. Therefore, we pay relatively low wages, if people relocate they can often live in relatively poor living conditions and accommodation (…) when you talk about safeguarding or when you talk about there are things that you may not naturally associate but that's a great example of our industry of maltreatment.

This was a significant reminder that maltreatment can be experienced across the football system, in places and with people who might not be expected to receive this behavior. The fragile working conditions of professional football (66) leave staff at the risk of also being maltreated, due to a surplus of other individuals who are willing to take their place. In this case, close attention is needed to monitor the potential for maltreatment to occur across the football system and for all individuals working within this environment to be mindful of the risks to them.

### 3.3 The signs and symptoms of maltreatment

Thus far, the participants discussed the result of the constituent behaviors of maltreatment coupled with the understanding of this concept in football. Aligned with this the participants also identified a range of signs and symptoms of maltreatment (see Table 3). The higher-order themes covered a range of psychological and physical signs and symptoms emotional effects, a damaged sense of self, impacted mental health, burnout, and disengagement.

**Table 3:** Signs and symptoms of maltreatment.

Example raw data code		Lower-order theme		General dimension
Suppressing emotions	→	Emotional effects	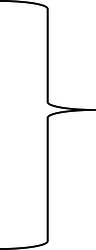	Signs and symptoms
Isolation and exclusion	→	A damaged sense of self		
Substance abuse and addiction	→	Impacted mental health		
Depersonalization	→	Burnout		
Lack of attendance	→	Disengagement		

#### 3.3.1 Emotional effects

One of the most common references made by the participants about the signs and symptoms of maltreatment was linked to emotional effects. On the more overt end of this continuum, Dave described a fear state experienced within the football context which mirrors other sports (72),

I've heard where adult players in high performance (…) they've come to a match and they're looking for the coach's car hoping that that particular coach isn't there that day. Then (…) they're pleased when the particular coach is not there because they're not gonna be shouting at them and, you know, getting on the backside. They're relieved (…) and then they go and play better.

This extract was indicative of both the maltreatment experienced in terms of shouting and criticism, as well as demonstrating how players can move from a position of preoccupation to relief. Within football beliefs persist that abuse, intimidation, and violence enforce authority and maximize the potential for winning (29, 72). Yet, Dave's account highlighted that the “win at all costs” view in football is flawed as players' performances improve once a perpetrator of maltreatment's presence is removed.

While the experiences shared by Dave highlighted an overt form of emotional effects, others pointed towards something more nuanced and subtle to observe, where the effects only appear later,

[The coach thinks] the players will listen to me now and will respect me if I scream and shout and throw a boot across the changing room. Well, we know they don't. We know that young people will completely block their ears off. Some will listen tentatively and be scared out of their wits and others will just switch off. So actually, it's not an effective use of communication at all. (Layla).

Here this participant demonstrated the layered nature of maltreatment as well as misguided beliefs in football around how respect is gained. In the moment, though some will display the emotional effects of fear, others more subtly suppress their emotions under the apparent guise of disengagement which may be theorized as a form of expressive suppression (73, 74) where individuals use active approaches to disengage from maltreatment. At a broader cultural level, Layla's account reflected something more concerning though in terms of the gradual process by which players become desensitized but then the resultant stress of this experience is exposed later in unmanaged emotions. This is problematic for the long-term mental health of these individuals (74). As Layla described, emotions may only be expressed in a dysregulated or angry fashion as the participants have not been allowed to express them productively (75).

#### 3.3.2 A damaged sense of self

Related to some of the emotional effects experienced resulting from maltreatment, the participants also alluded to the outcome of a damaged sense of self. Simon outlined the far-reaching impact of how maltreatment can impact on individuals in football,

I think it can be a horrendous knock to their confidence, your self-esteem, your identity, you've gone from being an established first-team squad member to now you're out in the cold, but you've still got to turn up and see these people every day. You've still got to interact, and you've still got to work with them. And then how does that affect your pecking order with your mates? You've gone from being the top player in your age group to, this guy's with the first team all day every day, now he's back with us. For somebody at such a vulnerable age that veneer of self-confidence is, it cracks like ice.

At the individual level, this account depicted a huge impact on the sense of self for those on the receiving end of maltreatment, with in this case, players' status being impacted such that they may become isolated, and possibly even embarrassed. This though, is coupled with potential feelings of humiliation where the nature of the club environment means that individuals must keep attending despite not playing and their relationships can become seriously affected.

Notably, reference was made by both the previous participant and others to an individual's self-esteem, given the global nature of this concept. Extending on research surrounding the legacies of abuse in sport (76, 77) Myles captured how the effects of maltreatment transcend the football environment,

Then it's going to have an effect on their self-esteem and then passes on to the rest of the life to the family and you know into the job at work that kind of thing, so I think it's something that you can't underestimate that side of it.

The pertinence that Myles gave to the point that maltreatment can impact others beyond the football environment was important to highlight. Grounded within findings with elite gymnasts (72), there is concern that players in particular, may need long-term psychological therapy as a result of maltreatment and may be at risk of clinical issues such as posttraumatic stress. Myles demonstrated that maltreatment can be particularly far-reaching and impactful, with the signs and symptoms of this behavior ricocheting into other contexts. Consequently, detecting maltreatment might not be directly observable in the football environment.

#### 3.3.3 Impacted mental health

Given the associated emotional effects of maltreatment and the resultant damage to an individual's sense of self, it is unsurprising that the participants discussed the mental health impact of this behavior. Nonetheless, the gravity of the impact of this behavior was particularly concerning with a range of externalizing symptoms presented including substance abuse, other forms of addiction, and most disturbingly of all, fatality.

There's that danger of escalating behavior. But if you are achieving your aims through those methods of maltreatment. You may think that's an effective way to undertake your business, but unfortunately, there could be a casualty to that. Either there or sometime in the future. I've probably been to 10 to 15 young people who have hung themselves on the back of the bedroom door… being there with a child's dead body, so it becomes very real to me of the potential consequences. Whereas if you're a football coach, you wouldn't have dreamt of being in that scenario, but maltreatment could lead to that. (Giles)

The experience of Giles is important to draw on here as they mirrored findings which suggest that various forms of maltreatment result in greater suicide attempts (78) and highlighted how fatality could occur with adults in professional football. These findings offer the potential to extend previous research (79) by shedding some light on why male adults may show signs of “male depression” and suicidal behaviors when they do not have a historical record of child abuse and neglect. Instead, it may be that they are maltreated within a particular context such as professional football, where these behaviors are not recorded. That then leads to these mental health outcomes. Moreover, Giles' account demonstrates that coaches may lack the necessary understanding or responsibility for their actions in relation to maltreatment and carry on with this behavior regardless, due to false beliefs about what constitutes success.

Though less severe in outcome, the participants also alluded to a range of other externalizing symptoms which were more common with the extant literature on maltreatment in sport (76, 77) such as various forms of addiction as well as internalizing symptoms such as disordered eating (72). Keeley, demonstrated this view whilst also highlighting a raft of addictions which might occur due to maltreatment,

You know, there will be the drink, the drugs, the gambling, the sex addiction, all that type of stuff. That's how it can impact but also massively on you know that mental health side of things.

It is also important to note that this participant couched these points within describing individuals' “behavior away from the club,” reinforcing that effects of maltreatment may be experienced in isolation and may be subtle to detect. Furthermore in highlighting externalizing behaviors Keeley may have provided a more specific reflection of the football culture where actions such as sexual addictions may be celebrated as part of an individual's masculine identity (30). Consistent with this Keeley also discussed the possibility for eating disorders. “Eating disorders is another one actually. We see that, we do see eating disorders. And not necessarily related to players who’ve had weight issues either.” Keeley's reference to those not having weight issues reinforced a sense that maltreatment can happen unexpectedly and the impact of it on mental health may not always be obvious to see. This was a point Robert reinforced, through discussing how maltreatment “might manifest itself in other ways, lack of sleep or irritability or feeling sick or anxious or missing training or whatever the case may be.” Some of the subtleties are worthwhile to stress as it may mean that these behaviors get lost, and maltreatment is in operation without being identified.

#### 3.3.4 Burnout

Although the participants often discussed the signs and symptoms of maltreatment at an individual level, the systemic risks because of this behavior were also evident in some accounts. This was most clearly expressed by Anna,

The [football] environment doesn't allow for that type of reflection because it is constant like there is, there is no let off whatsoever (…) Some of these guys work far too many hours, (…) they're accessible to parents 24 h a day. Often you know, there's no cut-off, there's very little boundaries. You know, that's a massive concern for me in terms of staff wellbeing.

This extract provided an important reminder about the breadth of maltreatment in that the participant discussed the effects on wellbeing across the staff group. It also re-emphasized that maltreatment is not a phenomenon experienced by coaches and players alone. Instead, this happens across football clubs and is governed by the expectations of the football environment more broadly. Thus, from a theoretical perspective, the encompassing tendencies of the total institution of professional football (80) create a relentless work culture within which wellbeing is de-prioritized. The outcome of this 24/7 culture is that individuals can leave football very early in their careers,

In support of Anna, the damaging cultural expectations of the football environment and key individuals within it were outlined by Cheryl,

I think there's a lot of burnout, there's a lot of when people are burned out and they're off on stress. Because I sit in the HR office, and I can hear the managers coming in and complaining about people that are off. I had one manager saying I don't believe her that's she on stress, I think she's just skiving, let's get rid of her (…).

Cheryl revealed a wider concern about the link between maltreatment and burnout in that individuals' feelings are not respected, and instead, they are accused of being off work for illegitimate reasons. Within this “captive world” (80, p. 15) all of football's individuals have to display the ideal identity to survive in this culture (11). This compounds the maltreatment of these individuals as they may then feel forced to return to work when they are not ready.

Considering the above this form of maltreatment it is not surprising that one of the common experiences of burnout, depersonalization, is evident,

And then the really extreme cases (…) this is a similar experience to my own, not from a playing capacity, but you start questioning who you are, because your identity is so wrapped up in what you do (…) What's the point in being here? (Simon).

Multiple layers were revealed here in terms of identifying maltreatment in football contexts. Simon illustrated how from a systemic perspective maltreatment can be experienced by various staff members, as well as players, whilst also demonstrating at the individual level how individuals lose their sense of worth and identity. This lost sense of identity appears to be symptomatic of the core elements of burnout syndrome such as emotional exhaustion and a feeling of depersonalization (81).

#### 3.3.5 Disengagement

Given the conceptualization of burnout raised by both the participants, as well as within research, proposes a reduced sense of accomplishment (81) it was unsurprising that the final sign and symptom of maltreatment in football was linked to disengagement. The findings gave the sense that the potential devaluation individuals experience from burnout resulting from maltreatment can lead to disengagement from their work in football. Worryingly the views expressed by the participants in this study align with findings from high-risk adolescent youths (82), whereby maltreatment leads to a form of disengagement coping. For some, such as Claire, disengagement was a clear marker of maltreatment,

I guess from a staffing point of view (…) if you start seeing somebody disengaging in work, in the club, if they're not coming in, if they're frequently unwell, if there's tension, I think you can always tell if there's an atmosphere or awkwardness, then I think those things would indicate that something was going on. But I don't know that people outside of the safeguarding, player-carey-type of remit would necessarily pick up on it as much. I think we're a little bit more tuned to think what else is going on, rather than they’re just being moody or they're being difficult or they're being lazy or whatever else somebody else might say.

Several layers were revealed here at both an individual, club and systemic level in relation to the signs and symptoms of maltreatment. On the individual level, a range of behavioral signs were identified which would first demarcate disengagement stemming from maltreatment. Though these signs would only be observable to key individuals within the organization such as safeguarding and player care staff. Claire's quote also revealed dangerous misconceptions about how disengagement might be perceived by others within the club. Thus, at this level, it suggests that potential maltreatment may be exacerbated, rather than addressed. If is the case this is a particular cause concern in relation to future aggressive acts which might follow, particularly as disengagement coping increases as a function of age (82). Despite the potential issues with disengagement coping, there appears to be a lack of understanding of the impact of maltreatment across the football system. Instead worrying views are digested by those who experience maltreatment, as Simon described,

This one player, in particular, the one with the phone call incident. He said he was actually, on reflection, further down the line, it helped toughen him up a bit, give him a thicker skin. He's had a real dip on confidence, but it feels like he's coming back and he's even more determined, more motivated. But for other people, you might have just continued on that downward trajectory and that can be quite a scary place I think.

Worryingly for the player concerned, it appears that maltreatment led them to initially disengage but then they perceived that this behavior may be to their benefit. This type of findings is representative of the worrying conflation of maltreatment with terms such as mental toughness and resilience in sport (25). As such, it pointed to the clear need to focus on this misunderstanding, as well as the other signs and symptoms of maltreatment and find effective means to address this behavior.

## 4 General discussion

The present study took a unique co-designed approach to understanding maltreatment in professional football through combining the expertise of those with experience of safeguarding in this context with an evidence-based approach to the findings. The results propose important considerations for how maltreatment is conceptualized in the football context and beyond. From a conceptual perspective, the present study provides an important extension to Stirling's (18) model of maltreatment in sport by highlighting the importance of adding banter to frameworks which broadly encapsulate wrongdoing. In partnership with those working within safeguarding roles in professional football, the findings also reaffirm a sense that bullying occurs within critical as well as non-critical relationships in football (8), which acts as a further extension of Stirling's (18) model. Furthermore, the present study demonstrates notable considerations for those working in football and other financially lucrative high-performing contexts about the potential for maltreatment to result from the commodification of individuals in a way which might not be seen in other sports. Specifically, professional football's reliance on the loan system to develop and trade players as well as its fragile working conditions may mean that individuals' welfare and wellbeing are jeopardized (66, 71).

Further to establishing important information about the constituents of maltreatment, the present study outlined important guidelines for spotting maltreatment, as well as its effects in terms of individuals' mental health and sense of self. While some emotional effects are noticeable and disengagement can be used as a form of coping, football's safeguarding personnel outlined a worrying trend towards the emotional impact of maltreatment being expressively suppressed (74), leading to a combustible reaction (75). The result is a potentially disastrous set of mental health outcomes ranging from suicide at the most extreme to a range of highly negative internalizing and externalizing symptoms (76, 77).

Last, the present study provided an important cultural overview of how the professional football environment may create ideal conditions for maltreatment to take place. In particular, the participants highlighted an environment where abuse and intimidatory acts are commonplace, leading to subordination on behalf of the recipients of these forms of maltreatment (11, 29, 31). The participants described how these overt forms of behavior, when coupled with a lack of knowledge and awareness of football's personnel, can lead to a grey area in relationships where maltreatment can thrive (14). This raised significant concerns around the duty of care being provided in this sport.

### 4.1 Applied implications, limitations and future directions

From a practical perspective, the present study highlighted some key implications. The participants alluded to greater efforts needing to be made to raise the level of education and awareness of maltreatment in professional football, as well as the need to develop programs which tackle the problematic culture of this sport. Specifically, football personnel need to be made aware of problematic behaviors which operate within potential grey areas of acceptability and be ready to challenge potential wrongdoing which may be experienced through actions such as banter. Additionally, football personnel need to be mindful that the signs and symptoms of maltreatment may not be obvious and may instead represent themselves in behavior such as disengagement which acts as a screen for potential mental health outcomes. More widely, football's governing bodies need to consider the working practices of this sport and how individuals' welfare can be better safeguarded through systems such as player loans.

Despite this study aided the understanding of maltreatment in professional football, it is important to acknowledge its limitations. The present study also did not provide further demographic information on the personnel involved in this research which may have limited the contextualization of the findings. Unfortunately, this was not possible as it may have jeopardized the anonymity of individuals who were part of the study. Similarly, although several different personnel in football were involved in the present study, it did not capture the views of football players and coaches. This is important to highlight in light of a range of recent research which has sought to empower the voices of athletes in relation to their maltreatment experiences (72) and players’ experiences of whistleblowing (23). Additionally, this may be seen as a limitation in light of research which has sought to empower end users' views of adult safeguarding (83). However, research has noted that football players (for example) may be apprehensive about sharing their views about forms of maltreatment and including a broader range of personnel may be beneficial in developing a stronger organizational focus in the broad area of safeguarding (23). Consistent with this point, although the present study provided important findings in relation to a critical element of safeguarding (e.g., understanding maltreatment) it does not discuss how a future safeguarding program may be operationalized in terms of its delivery. Therefore, future research may wish to explore recommendations for the design of safeguarding programs, with those staff invested in this area in both professional football and sport more widely.

## Data Availability

The datasets presented in this article are not readily available because The participants did not give their consent for the data to be shared publicly. Requests to access the datasets should be directed to j.newman@shu.ac.uk.
